# Minimum load threshold in resistance training: insights into muscle metabolism, excitation, and fatigue across the repetition continuum

**DOI:** 10.7717/peerj.20909

**Published:** 2026-03-12

**Authors:** Alessandro L. Colosio, Brecht D’hoe, Jan G. Bourgois, Jan Boone

**Affiliations:** 1 Laboratoire Interuniversitaire de Biologie de la Motricité, Université Jean Monnet, Saint-Etienne, Loire, France; 2 Department of Movement and Sports Sciences, Universiteit Gent, Ghent, Belgium

**Keywords:** Strength training, Exercise prescription, Near-infrared spectroscopy, Electromyography, Maximal voluntary contraction, Critical power

## Abstract

**Background:**

High external loads are typically prioritised in resistance training (RT), but low-load RT (<50% 1 repetition maximum (RM)) has gained attention for its practical and physiological advantages. While mechanical tension and metabolic perturbation are key drivers of hypertrophic adaptations, the mechanisms underlying effective thresholds for low-load RT remain unclear.

**Purpose:**

To investigate the physiological responses, fatigue, and recovery during concentric-only RT performed to failure across a spectrum of % 1RM, with particular focus on the potential relevance of the critical load (CL) concept as a physiological boundary.

**Methods:**

A total of 12 participants (six women, moderate RT experience) performed exhaustive unilateral leg extension at 10%, 30%, 50%, 70%, 90% 1RM. Muscle deoxyhaemoglobin and muscle excitation were measured respectively with near-infrared spectroscopy and electromyography (EMG). Heart rate, blood lactate, and rate of perceived exertion were measured as indicative of whole-body responses. Maximal voluntary contractions (MVC) were executed before and up to 30 min after each protocol. CL was calculated based on the load used and the time required to reach muscle failure. Responses between different % 1RM were compared using 1-way and 2-way repeated measures analyses of variance, followed by *post-hoc* analyses. Repeated-measure correlations were calculated between fatigue accumulation and the main physiological variables.

**Results:**

The 30%, 50%, 70%, 90% 1RM protocols induced muscle failure and similar levels of local and whole-body metabolic perturbation, while the 10% did not lead to failure and induced lower metabolic perturbation. Muscle excitation upon exhaustion increased with increasing external loads and did not lead to common EMG levels between % 1RM. A total of 30% and 50% 1RM protocols caused significant MVC reduction *vs*. baseline, and fatigue was moderately correlated with metabolic markers. CL was detected at a load corresponding to the 31.7 ± 11.9% 1RM.

**Conclusion:**

The characterisations of the acute physiological responses to RT across light to heavy loads performed in this study provide potential insight for determining the minimal load threshold in RT and suggest a potential proximity between the CL and the load associated with blood flow occlusion during contraction.

## Introduction

Resistance training (RT) encompasses a diverse range of exercise modalities aimed at improving strength, hypertrophy, and muscle endurance through varying equipment and strategies, such as free weights, machines, or body weight. Historically, RT has been closely associated with high external loads due to cultural influences that shaped early guidelines prioritising heavier intensities to optimize hypertrophic adaptations and strength gains (Ratamess et al., 2009; Kraemer et al., 2017). Although classical recommendations often suggest moderate to heavy loads (60–80% of 1 repetition maximum (1RM)) for hypertrophy and even higher intensities for strength gains, accumulating evidence challenges the necessity of such strict prescriptions (*e.g*., Schoenfeld et al., 2017, 2021).

In the context of hypertrophy, low-load RT (<50% 1RM) has gained attention as a viable alternative for populations unable or unwilling to engage with traditional heavy RT, such as clinical or older populations (Schoenfeld, 2013; Lasevicius et al., 2018; Weakley et al., 2023). Beyond its practical advantages (*e.g*., requiring less equipment or external load), the effectiveness of low-load RT suggests that achieving a certain level of metabolic stress within the muscle may be essential to stimulate hypertrophic adaptations. For instance, while mechanical tension is regarded as the main player in inducing muscle hypertrophy (Wackerhage et al., 2019) it remains uncertain whether such tension must arise primarily from high external loads (*i.e*., mechanical stress due to force production) or can also emerge through sustained effort and fatigue accumulation during low-load contractions. A distinction that relates also to the broader question of whether similar hypertrophic outcomes from low- and high-load RT are underpinned by shared or distinct mechanisms (Ogborn & Schoenfeld, 2014). Specifically, it is still debated whether metabolic perturbation related to intramuscular changes in energy provision, metabolite accumulation, and oxygenation status is a necessary cofactor in this adaptive process. Intramuscular lactate accumulation has been associated with hypertrophic signalling and may act as a proxy for the level of energetic stress achieved during exercise (Lawson et al., 2022). Thus, a given threshold of metabolic disturbance might be required to complement or potentiate the effects of mechanical tension in promoting muscle growth, particularly in low-load RT.

Research on acute responses to a single session (Burd et al., 2010) or hypertrophic adaptations following training periods (Lasevicius et al., 2022) suggests the minimum load threshold of low-load RT is around 20–30% of the 1RM (Schoenfeld, 2013; Lasevicius et al., 2018; Weakley et al., 2023). This load marks the point eliciting sufficient mechanical and metabolic stimuli, but also coincides with the intramuscular pressure required to induce local blood flow occlusion during contraction (Fliss et al., 2025), which might accelerate fatigue and motor units recruitment (Loenneke, Wilson & Wilson, 2010). A less explored aspect is that this threshold roughly overlaps with a muscle’s critical load (CL), the local and systemic boundary separating sustainable and unsustainable intensity domains (Monod & Scherrer, 1965). While applying the CL concept to RT is difficult due to technical and practical reasons, it could offer a framework to interpret RT responses in terms of local exercise intensity domains and their distinct physiological stimuli (Colosio et al., 2021; Inglis et al., 2024). In this context, monitoring local metabolic perturbation and muscle excitation across the full repetition continuum under standardised, rhythmic contraction patterns (*i.e*., endurance-like), may help position different RT loads within the framework of local exercise intensity domains.

While higher loads recruit a greater number of muscle fibres at the onset of exercise, lower loads may progressively recruit additional fibres as active fibres fatigue, balancing the decline in force output (Henneman, Somjen & Carpenter, 1965). Previous EMG-based studies comparing high- and low-load RT have generally failed to demonstrate muscle activation levels at the end of the working sets (Cook, Murphy & Labarbera, 2013; Akima & Saito, 2013; Schoenfeld et al., 2014; Jenkins et al., 2015; Morton et al., 2019), but these protocols typically employed relatively short between-repetitions rest time (*e.g*., 1:1 s concentric:eccentric in Schoenfeld et al. (2014) and from 1:1:1 to 3:1:3 s concentric:pause:eccentric in Morton et al. (2019)). This structure may have caused early fatigue in the already active muscle fibres, particularly at lower loads, limiting the time available for the progressive recruitment of additional motor units. As previously suggested in endurance contexts, reduced contractile efficiency over time may impair force production before full motor unit activation is achieved (Grassi, Rossiter & Zoladz, 2015).

Another key consideration is the measurement of fatigue, typically defined as a decline in mechanical force or power (Twomey et al., 2017). Low-load RT often requires prolonged submaximal efforts to failure, and can induce significant localised fatigue due to the high number of repetitions needed to reach a sufficient training stimulus when external load is reduced (Behm et al., 2002; Weakley et al., 2023). While this is particularly relevant as greater repetition volumes are associated with increased fatigue accumulation (Behm et al., 2002), comprehensive evaluations of fatigue across the full % 1RM spectrum remain limited, partly due to variations in RT configurations (*e.g*., whole-body *vs*. isolated exercises), differences in fatigue mechanisms related to muscle mass involvement, and inconsistencies between gold-standard fatigue measures (*e.g*., maximal voluntary contraction (MVC)) and RT-specific protocols.

Given these considerations, unilateral leg extensions could provide a standardised and reproducible model to study RT while overcoming many of the methodological challenges associated with more complex, multi-joint exercises, yet still engaging a large muscle group. The quadriceps muscle provides a controlled environment for precise measurements of fatigue and physiological responses, enabling the application of advanced methodologies such as near-infrared spectroscopy ((NIRS) (Grassi & Quaresima, 2016)) and electromyography ((EMG) (Farina, 2006)). These techniques allow simultaneous insights into oxygenation and motor unit recruitment, offering a detailed understanding of the interplay between metabolic perturbations, mechanical tension, and the underpinnings of fatigue.

Despite increasing interest in low-load RT, it remains unclear how metabolic stress, neuromuscular activation, and fatigue interact across different loading conditions. A systematic comparison across the full spectrum of intensities is currently lacking. Specifically, it is unknown whether the convergence in muscle excitation and deoxygenation at task failure occurs uniformly across loads, and how this relates to the degree of mechanical fatigue incurred. This study aimed to address these gaps by applying a controlled concentric-only leg extension protocol across a wide range of intensities, combining NIRS and EMG measurements with post-exercise fatigue assessments. It was hypothesised that: (1) all protocols would induce muscle failure, with completion times increasing as external load decreased; (2) progressive recruitment and metabolic perturbation would result in comparable levels of muscle excitation and deoxygenation at task failure; (3) fatigue would be more pronounced following low-load RT, due to the higher number of repetitions performed.

## Materials and Methods

### Ethical approval and data availability

All procedures performed in this study were in accordance with the 1964 Helsinki Declaration and its later amendments or comparable ethical standards. The subjects were fully informed of any risks and discomfort associated with the experiments before giving their written consent to participate in the study. Ethical approval was obtained from the ethical committee of the Ghent University Hospital (Ghent, Belgium) (reference: ONZ-2023-0308; B6702023000526). The raw data supporting the findings of this study are available as Supplemental Material.

### Participants

A total of 12 healthy active participants (six men, six women, 21.8 ± 1.8 years, 70.2 ± 10.8 kg, 177.0 ± 9.7 cm, estimated maximal oxygen consumption: 49.8 ± 5.5 ml·min^−1^·kg^−1^ (Jackson et al., 1990)) participated in this study. Inclusion criteria were age between 20 and 35 years, being generally healthy and free from any recognised disease, and being engaged in RT for at least 2 months. Exclusion criteria were smoking and any condition that could influence the physiological responses during training, including pathologies or previous injuries to the lower limbs that could impact testing.

### Protocol

**Figure 1:**
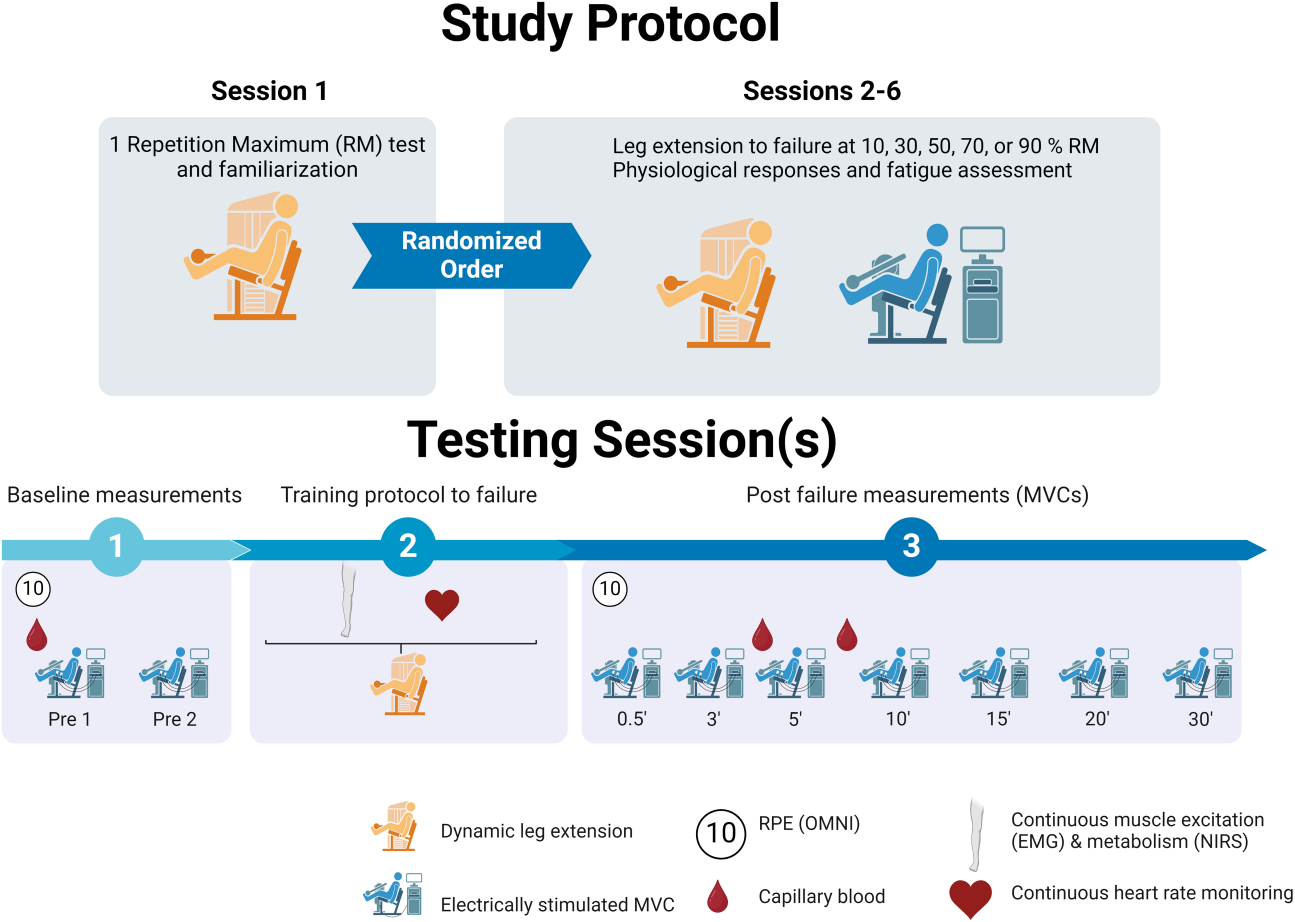
Protocol. Above: schematic representation of the study protocol; below: structure and measures of testing sessions from 2 to 6. Figure credit: BioRender.com.

All the experimental procedures took place at the Department of Movement and Sports Sciences of the University of Ghent (Belgium) in an environmentally controlled laboratory (~22 °C, 55–65% relative humidity). Subjects visited the laboratory at the same time of the day (±1 h) on six different occasions over a period of 6 weeks (Fig. 1). The first session consisted of a general familiarisation and instruction session, in which the individual’s 1RM of unilateral leg extension was determined. The following five experimental sessions were executed on different days in randomised order and consisted of sessions of a single set of unilateral leg extension (Selection 900; Technogym, Cesena, Italy) performed until muscle failure with the dominant leg and an external load corresponding respectively to 10%, 30%, 50%, 70%, or 90% of the 1RM. Before and after each protocol, maximal strength levels of the exercising leg were assessed during MVCs of the quadriceps performed on an isokinetic dynamometer set in isometric mode (Biodex System 3 Pro; Biodex Medical Systems, Shirley, NY, USA) and placed near the leg extension device. The optimal sit position of both the leg extension and the dynamometer was recorded for each participant during the first visit to the laboratory and maintained equal during all testing sessions. Muscle failure during leg extension exercise was determined as a drop of at least 10% from the initial full range of motion with full knee extension for more than two consecutive repetitions despite strong verbal encouragement (*i.e*., participants were stopped at the 3^rd^ failed repetition), and was monitored continuously using the displacement measured by a linear encoder (Tendo Power; Tendo Sports Machines, London, UK) connected to a personal computer. All participants were instructed to avoid caffeine consumption and physical activity, respectively for at least 8 and 24 h before each testing session. Each testing session was separated from the others by a minimum of 3 days (ideally, participants were tested once per week) and after participants self-reported a feeling of complete recovery and a score of 0 pain at the quadriceps level on a visual analogue scale (Williamson & Hoggart, 2005). This procedure was explained to the participants after each testing session. Moreover, they were instructed to follow the same standardised meal, including 2 g of low glycaemic index carbohydrates per kg of body weight and 0.5 L of water 2 h before testing.

### One repetition maximum test

After the preliminary procedures (*e.g*., fitting of leg extension position, explanation of the procedures, *etc*.), participants performed a general warm-up including 5 min of light cycling, 15 bodyweight squats, and 20 repetitions of bilateral leg extension with an external load of 10 kg. Thereafter, they performed three sets of respectively 10, 6, and 3 repetitions with progressively increasing weights, to reach an external load that could allow them to perform no more than 12 repetitions during an exhaustive set as proposed in the method by Brzycki (1993). The first of these sets was performed by all participants with a fixed weight of 20 kg, while the weight for the other two sets was increased by 5 to 15 kg based on the perceived exertion reported on an OMNI scale (Robertson et al., 2003). The targeted predetermined exertions for these three sets were “somewhat easy”, “somewhat hard”, and “hard”, corresponding to scores of 3-4, 5-6 and 7-8. Finally, weight was further increased by 5 to 10 kg for a single set in which participants had to perform the maximum number of repetitions possible under strong verbal encouragement. The allowed rest during this protocol was 3 min between each set. All participants remained under the target of 12 repetitions, with a mean of 7.3 ± 2.4. The so obtained combination of weight and performed repetitions was used to extrapolate the 1RM while avoiding exposure to maximal external load, and to calculate the loads for the sessions (*i.e*., 10%, 30%, 50%, 70%, 90% 1RM). Participants were seated with a knee angle of ~ 90° (flexion) and a trunk-thigh angle of 100°. This position was also maintained during the following visits.

### Leg extension at different % 1RM

After the 1RM estimation, participants completed 1 set of unilateral leg extension to muscle failure at five different % of the 1RM (*i.e*., 10%, 30%, 50%, 70%, 90%). A schematic representation of these testing sessions is presented in the bottom panel of Fig. 1. During the leg extension tests, participants adhered to a set rhythm of one repetition every 4 s, regulated by a metronome. This predetermined frequency, established during the pilot phases of the study, aimed to ensure consistent pacing across varying loads. The goal was to prevent confounding factors, such as excessively frequent contractions at lower loads leading to potential intermittent ischemia, artificially long contraction time (compared to what participants would naturally do for a given load) or extended rest intervals at higher loads. Participants were instructed to perform “concentric-only” repetitions and to fully extend the knee with a fast and complete concentric contraction at each repetition. The weight was then dropped back to the start position (*i.e*., slam down) after completion of the concentric phase. No attempt was made to standardise the concentric duration across loads, all tests were continuously supervised to ensure that participants maintained a consistent, visually even rhythm throughout the set. Any slowing in movement speed due to fatigue was expected and not corrected. A predetermined maximal duration of 30 min (*i.e*., 450 repetitions) was chosen to stop the exercise as indicative of sustainable exercise (Pogliaghi et al., 2023). Participants were continuously supervised during the whole duration of the protocols and verbally encouraged to reach maximal effort at any time they showed signs of approaching fatigue.

Muscle deoxyhaemoglobin (HHB) of the *vastus lateralis* (VL) was evaluated in microcirculation using a quantitative frequency-domain near-infrared spectroscopy system (OxiplexTS; ISS, Champaign, IL, USA) that provided continuous measurement (sampling frequency 1 Hz) of the absolute concentration (μM) of HHB as previously described (Colosio et al., 2021). After shaving, cleaning and drying the skin area, the NIRS probe was positioned longitudinally on the belly of the VL muscle ~15 cm above the patella (knee completely extended), attached to the skin with a bi-adhesive tape and secured with elastic bandages around the thigh to avoid undesired moves or light infiltrations. The device was calibrated before each test following the manufacturer’s recommendations, which include a warm-up period of at least 30 min to ensure thermal and signal stability. Calibration was then performed using optical calibration phantoms with known absorption and scattering properties, allowing the system to adjust its internal reference for accurate measurement of tissue optical coefficients. The mean adipose tissue thickness under the probe location was 7.7 ± 4.1 mm, with 2 participants exceeding the ideal limit value of 8 mm (Stuer et al., 2024). To account for individual variability in signal strength and absolute magnitude, HHB was expressed as percentage changes from baseline (see Data Analysis).

Surface EMG signals of the VL, *vastus medialis* (VM), and *rectus femoris* (RF) muscles were continuously recorded using a wireless system (1,500 Hz; ZeroWire; Noraxon, Scottsdale, AZ, USA). A pair of surface Ag/AgCl electrodes (Blue sensor, Ambu®, Ballerup, Denmark) was attached to the skin with a 2-cm inter-electrode distance as previously described (Colosio & Pogliaghi, 2020; Colosio et al., 2021). The electrodes were placed longitudinally with respect to the underlying muscle fibre arrangement, according to the recommendations for Surface EMG for non-invasive assessment of muscles (Hermens et al., 2000). Before electrode application, the skin was shaved, gently abraded, and cleaned with alcohol to minimise impedance. Semi-permanent ink marks allowed consistent re-positioning of the electrodes between sessions. The EMG transmitters connected to the electrodes were well secured with adhesive tape to avoid movement-induced artefacts.

To determine baseline strength level, fatigue and recovery of the leg performing the leg extension protocol, 5-s MVCs in combination with the interpolated twitch technique were performed before and after each protocol (2 MVCs with 3 min rest before the leg extension protocol, immediately after muscle failure, and at minutes 3, 5, 10, 15, 20 and 30, Fig. 1). Participants were seated with a knee angle of 90° and a trunk-thigh angle of 100°, while the position was secured by two shoulder harnesses. Single electrical stimuli (1-ms duration) were delivered to the femoral nerve using a constant-current stimulator (DS7A; Digitimer Ltd., Hertfordshire, UK) and self-adhesive electrodes (10 mm diameter). The cathode electrode was secured on the inguinal triangle, while the anode rectangular electrode was placed on the gluteal fold. The first MVC of each testing session was preceded by a familiarisation phase in which the intensity of the electrical stimulus was determined by progressively increasing the current until a maximum resting twitch response was generated. Initial intensity was set at 20 mA and increased with increments of 20 mA until a plateau in the contractile response was reached. This current was then increased by a further 20% to ensure supramaximal stimulation. During the experimental sessions, singlet stimuli were given during the plateau of the MVC (superimposed twitch) and 2 s after MVC to test the voluntary activation (control twitch) (Shield & Zhou, 2004). Participants received verbal encouragement throughout the MVC. On average, the first MVC after muscle failure required 33 ± 6 s to be executed.

Heart rate (HR) was monitored continuously (H10 Sensor; Polar, Kempele, Finland) throughout the whole protocol. Blood samples (20 μl) were drawn from the fingertip to measure blood lactate concentration ([La^−^]) at baseline upon arrival at the laboratory, and at the third and fifth minute after muscle failure, and were immediately analysed using an enzymatic-amperometric technique (Biosen C-Line; EKF Diagnostics, Kempele, Germany).

Finally, perceptual responses to exercise were monitored using a 0–10 rating of perceived exertion OMNI scale (Robertson et al., 2003). The scale was displayed to the participants at baseline, after muscle failure, and after the completion of the first MVC to avoid any possible delay in the fatigue assessment. When reporting this rate, participants were asked to refer to the leg extension protocol only.

### Data analysis

The raw EMG signals were rectified and smoothed using a fourth-order band-pass Butterworth digital filter with a frequency range set between 20 and 500 Hz. Root mean square (RMS) was calculated every second from the raw signal and was used as an index of the total muscle excitation for VL, VM, and RF (Vigotsky et al., 2018). The maximum RMS recorded during pre-MVCs was used to normalise the response during the leg extension protocols performed on different days, while HHB was normalised as a % increase *vs*. the 2 min baseline recorded before the leg extension protocol. Thereafter, the RMS signals and the NIRS-derived HHB response during each exercise session were time-aligned with the onset of exercise. For a given repetition cycle of 4 s, RMS and HHB were respectively considered as the peak RMS and the mean HHB values recorded. These durations were chosen to account for the different dynamics of the two signals and the short duration of the protocols at the highest % 1RM. The peak response in muscle metabolism and excitation was defined as the maximum values of HHB and RMS reached during the last minute of exercise of the leg extension, while the slopes of increase of HHB and RMS were calculated over the full duration of each protocol by determining the coefficient of the linear regression line fitted to the progression of each signal across repetitions, representing the average change in signal per repetition. In the same time window, the peak in heart rate response was also detected as the maximum value recorded.

Biodex data were visually inspected, and MVC torque was considered as the peak torque attained during the 5-s MVC. The baseline force for a given day was considered as the value reached during the two MVCs before the leg extension protocol. The peak torque in the post-exercise singlet was used to assess voluntary activation (VA). VA% was calculated as:

VA% = [1 – (superimposed twitch/control twitch)] × 100 (Shield & Zhou, 2004)

In the case that the superimposed twitch was not delivered at the peak force, a correction was applied using the force at stimulation:

VA% corrected = [1-((superimposed twitch × torque at stimulation/maximal torque)/control twitch)] * 100 (Strojnik & Komi, 1998)

Blood lactate accumulation was calculated as the difference between the highest [La^−^] value recorded after muscle failure and baseline [La^−^].

Finally, participants’ CL was calculated to identify the boundary between sustainable and unsustainable exercise based on the load used and the time required to reach muscle failure. Tests in which muscle failure was not reached (*i.e*., 10% and 30% in six participants) and those of excessively short duration (*i.e*., 90%) were excluded, as the hyperbolic model requires a minimum range of time points to fit the curve reliably. Individual CL values were then calculated using three mathematical models: 2-parameter hyperbolic, linear, and linear 1/time, applying a best-fit approach (Caen et al., 2019).

### Statistical analysis

Statistical analysis was performed using Origin Pro 2024 (OriginLab Corporation, Northampton, MA, USA). First, normality within conditions and sphericity were checked, respectively using the Shapiro-Wilk test and Mauchly’s test. Data are presented as mean ± SD. One-way repeated-measure ANOVA was performed to assess differences in peak responses of VL, VM, RF (*i.e*., RMS and HHB), RPE, HR, and [La^−^] accumulation and HHB and RMS slopes (only for VL) between different % 1RM, while *post-hoc* analyses were performed using the Holm-Sidak method. Two-way repeated-measures ANOVA with Greenhouse-Geisser correction, followed by Holm-Sidak *post-hoc* analysis, was employed to evaluate differences between pre-MVC and post-muscle failure MVCs and their respective VA%.

The individual’s return to the MVC baseline was determined by identifying the time point (*i.e*., MVC) at which the force value returned to baseline levels as defined by the upper boundary of the 95% confidence intervals (CI) of the pre-MVCs. Furthermore, differences in the total kilograms performed (*i.e*., kg × number of repetitions executed) and delta values between MVC and VA% (*e.g*., values recorded during the first MVC after muscle failure and pre-MVC) among different % 1RM protocols were examined using one-way repeated-measures ANOVA with Greenhouse-Geisser correction, followed by Holm-Sidak *post-hoc* test.

To ensure the reliability of the collected data, intra-class correlation coefficients (ICCs) were calculated for the MVC pre-testing values and absolute baseline HHB values before each testing session.

To test the possible relationships between the slopes of RMS and HHB at different % 1RM and between fatigue, recovery, and the other measured variables, repeated measures correlation coefficients (Bakdash & Marusich, 2017) were calculated between the drop in MVC %, the delta in VA%, the time to return to pre-MVC and the total kg lifted, the peak responses of VL, VM, RF (RMS and HHB), RPE, HR, and [La^−^]. These correlations and the ICCs calculation were performed using Python 3.13.

Justification for the sample size was determined *a priori* based on a power analysis using G*Power (version 3.1). For the primary outcome variable MVC, an expected small-to-medium effect size (f = 0.22) was justified by the study of Behm et al. (2002), which reported substantial reductions in MVC (up to 21.4%) following muscle contractions performed with high and medium loads (*i.e*., 5RM to 20RM). An assumed correlation of r = 0.80 among repeated measures was based on findings by Kadlec et al. (2021), who reported excellent reliability (ICC = 0.86–0.95) for isometric MVCs in controlled conditions. Using these parameters, along with an alpha level of 0.05 and power of 0.80, the required sample size was calculated as 10 participants. Statistical significance was accepted when *p* < α.

## Results

The estimated 1RM of unilateral leg extension from the initial 1RM estimation sessions was 52.9 ± 12.9 kg (men 63.3 ± 5.71 kg and women 42.5 ± 8.80 kg), positioning them in a moderately resistance-trained status, meeting the necessary criterion for assessing acute physiological responses while minimizing confounding factors such as variability in performance across sessions (MVC and 1RM values aligned with moderately trained status; Cannon & Marino, 2010; Maden-Wilkinson et al., 2020).

**Figure 2:**
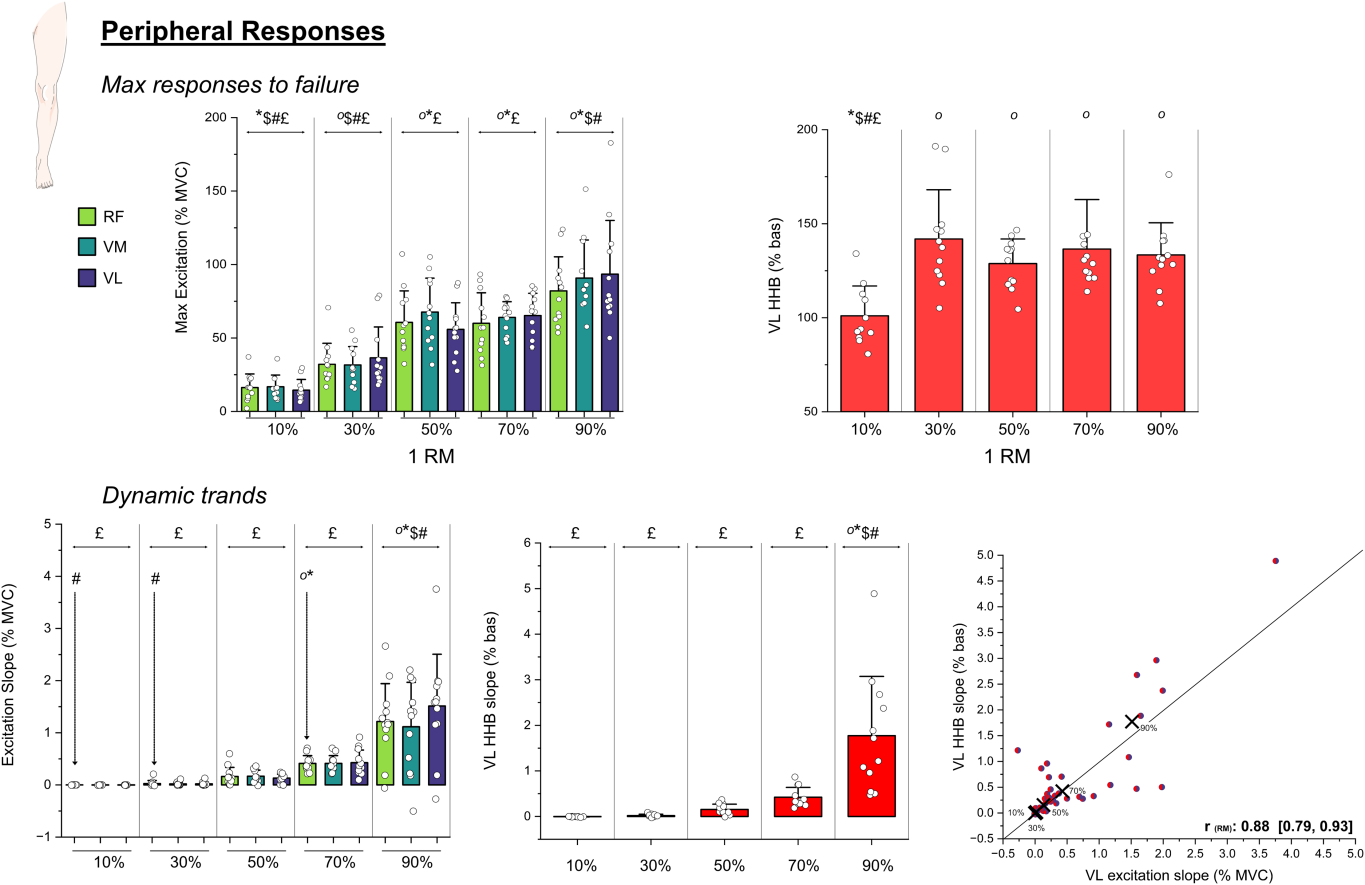
*Peripheral responses*. Above: individual peripheral muscle excitation ((RMS) at the vastus lateralis (VL), vastus medialis (VM) and rectus femoris (RF)) and deoxyhaemoglobin ((HHB) at the VL only) reached upon muscle failure by participants during the five protocols at different % of the 1 repetition maximum (1RM); below: individual dynamic slope increases of RMS (VL, VM and RF) and HHB (VL only) during the leg extension during the five protocols at different % 1RM, bottom right: individual slopes of HHB expressed as a function of the VL RMS slopes are presented together with the repeated measured correlation of these dataset. Histograms represent mean ± SD. Symbols indicate statistical significance *vs*.: ° = 10%, * = 30%, $ = 50%, # = 70%, £ = 90%. Please note that 10% represents the value at 30 min, not failure. This image has been prepared using freely available icons from https://bioicons.com/?query=leg.

The selected weights for the leg extension protocols were: 10%: 6.7 ± 1.6 kg, 30%: 15.9 ± 3.5 kg, 50%: 26.3 ± 6.7 kg, 70%: 37.3 ± 9.1 kg, 90%: 47.7 ± 11.4 kg. Participants performed respectively 10%: 450.0 ± 0.0 repetitions, 30%: 291.8 ± 175.6 repetitions, 50%: 42.9 ± 32.1 repetitions, 70%: 13.2 ± 3.8 repetitions, 90%: 5.6 ± 2.2 repetitions. CL was determined at 15.7 ± 4.3 kg (corresponding to the 31.7 ± 11.9 % 1RM), with an average difference between models of 0.8 ± 1.0 kg. No participant reached muscle failure during the 10% protocol, while there was a high variation in repetitions performed at 30%, with six participants (50%) not reaching muscle failure at this load. This difference between % 1RM protocols was reflected by HHB as measured by NIRS upon muscle failure/end of the exercise, with the 10% showing lower HHB values than all the other protocols (*p* < 0.0001, Fig. 2) and the 30% protocol displaying the highest variability in the responses (Fig. 2). On the other hand, RMS in the VL, VM, and RF, did not display the same level of muscle excitation upon muscle failure, but rather progressively higher % excitation at increasing external load (*i.e*., % 1RM, *p* < 0.0001 for all three muscles, Fig. 2). In terms of dynamic responses, there was a main effect of the % 1RM on the slopes of both HHB and RMS (both *p* < 0.0001, Fig. 2), with the 90% protocol displaying significantly higher slopes for all these parameters. Moreover, RMS and HHB slopes of the VL across different % 1RM were highly correlated (*p* < 0.0001, repeated measure r: 0.88, Fig. 2).

**Figure 3:**
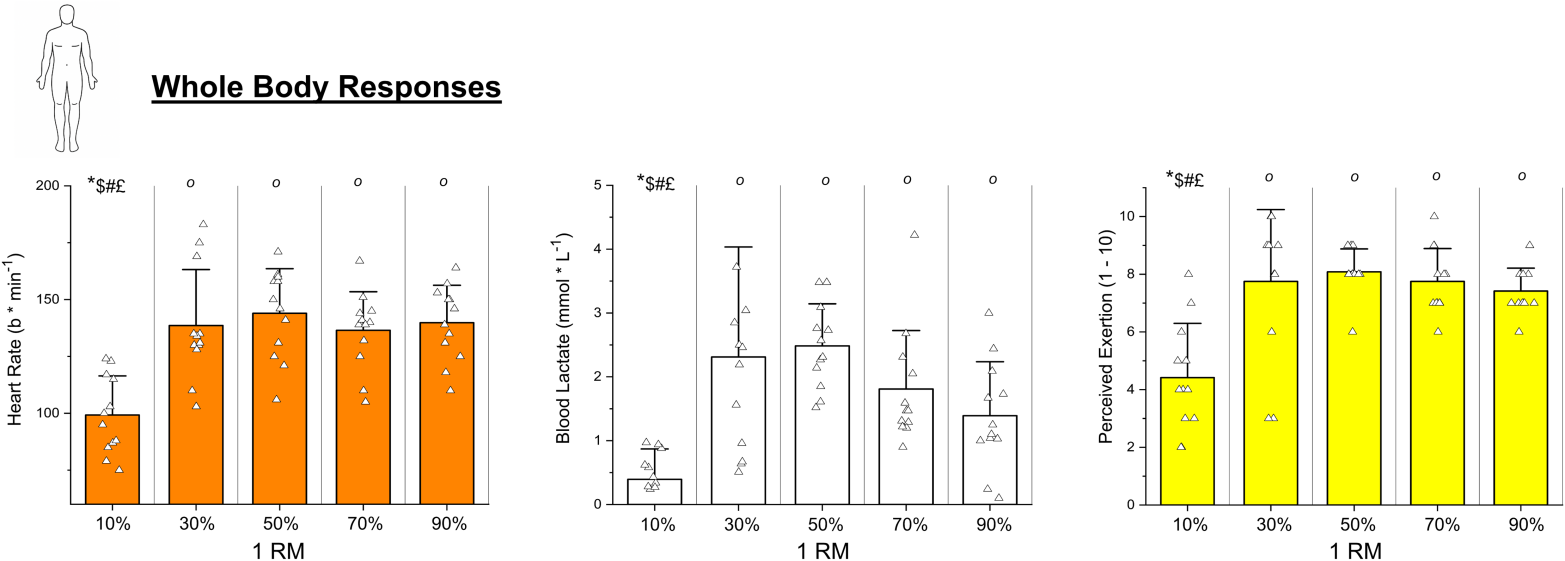
Whole body responses. Individual values of heart rate, blood lactate accumulation, and rate of perceived exertion (OMNI scale) reached upon muscle failure by participants during the five protocols at different *% of the 1 repetition* maximum (1RM). Histograms represent mean ± SD. Symbols indicate statistical significance *vs*.: ° = 10%, * = 30%, $ = 50%, # = 70%, £ = 90%. Please note that 10% represents the value at 30 min, not failure.

Whole body markers of strain (*i.e*., HR, [La^−^], RPE) reflected similar trends to what was reported for HHB, with 10% showing significantly lower values from the other protocols (all *p* values < 0.0001, Fig. 3).

**Figure 4:**
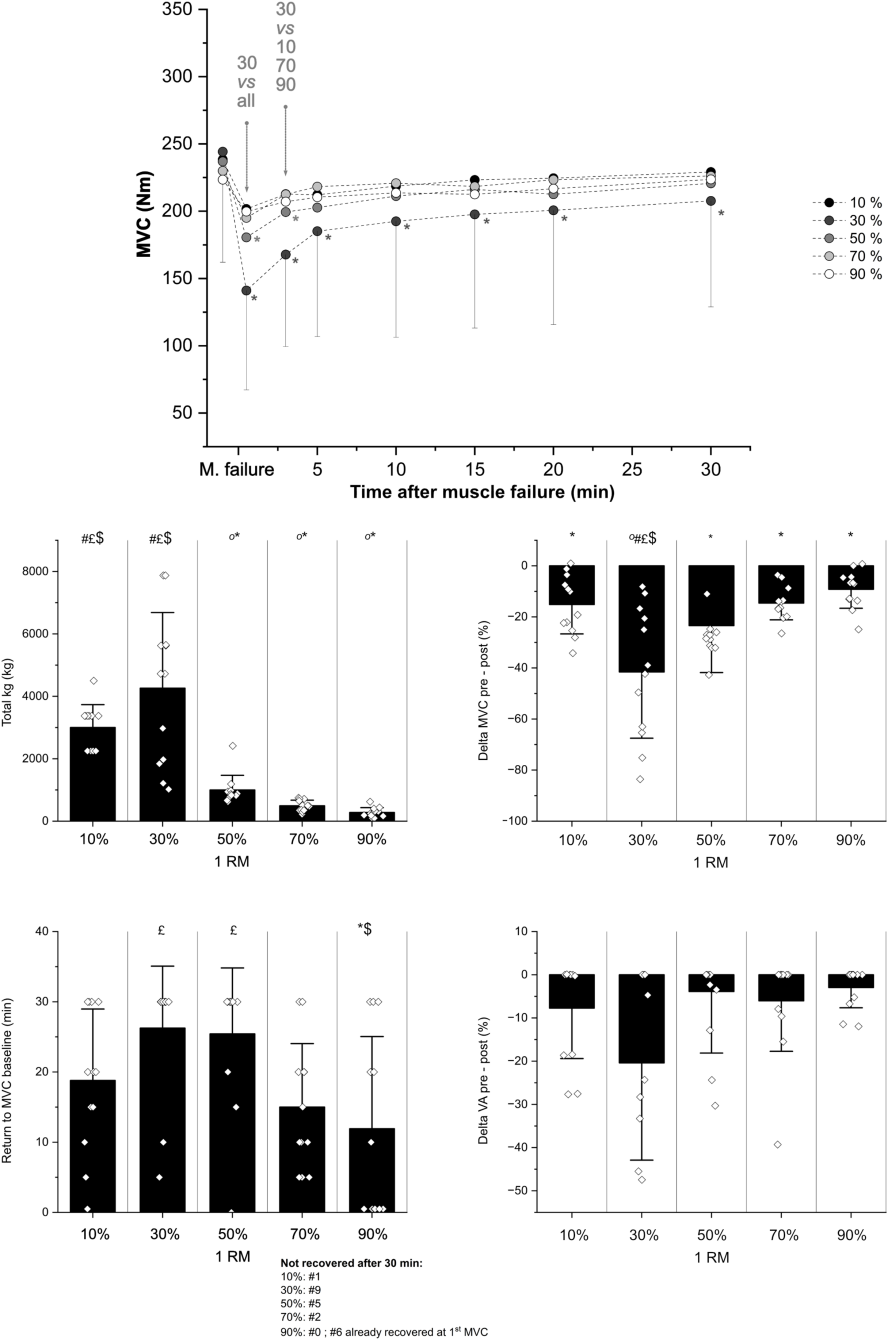
Fatigue. Above panel: the mean ± SD values (only 30% for clarity) of maximal voluntary contraction (MVC) before and after the leg extension protocols are reported in the panel above. *indicates statistical difference *vs*. pre-MVC at a given time point. Statistical differences between protocols for a given time point are represented by the grey words. Four panels below: individual values of total kg lifted, time to return to pre-MVC and deltas between baseline and the first MVC after muscle failure respectively for force values and voluntary activation in the five training protocols at different Histograms represent mean ± SD. Symbols indicate statistical significance *vs*.: ° = 10%, * = 30%, $ = 50%, # = 70%, £ = 90%.

In terms of fatigue, the two-way RM ANOVA revealed a significant main effect of time for MVC (*p* < 0.0001), as well as a significant time × trial interaction (*p* = 0.01), indicating that force output varied over time and that recovery dynamics differed across loading conditions. *Post-hoc* comparisons showed that MVC was significantly reduced from baseline for the 30% and 50% 1RM trials, and this reduction remained significant respectively up to 30 and 3 min post-failure. For the other conditions, no significant differences from baseline were detected at the group level after failure. In contrast, for voluntary activation VA%, the ANOVA showed no time × trial interaction (*p* = 0.19). To further explore immediate neuromuscular fatigue, delta values between pre- and post-exercise were analysed. Delta values differed significantly between trials for both MVC (*p* < 0.0001) and VA% (*p* = 0.02), as displayed in Fig. 4. Finally, the time required to return to baseline MVC values also differed significantly across protocols (*p* = 0.01). All participants recovered within 30 min after the 90% 1RM trial, while a lack of recovery was observed in several individuals at lower intensities (Fig. 4).

**Figure 5:**
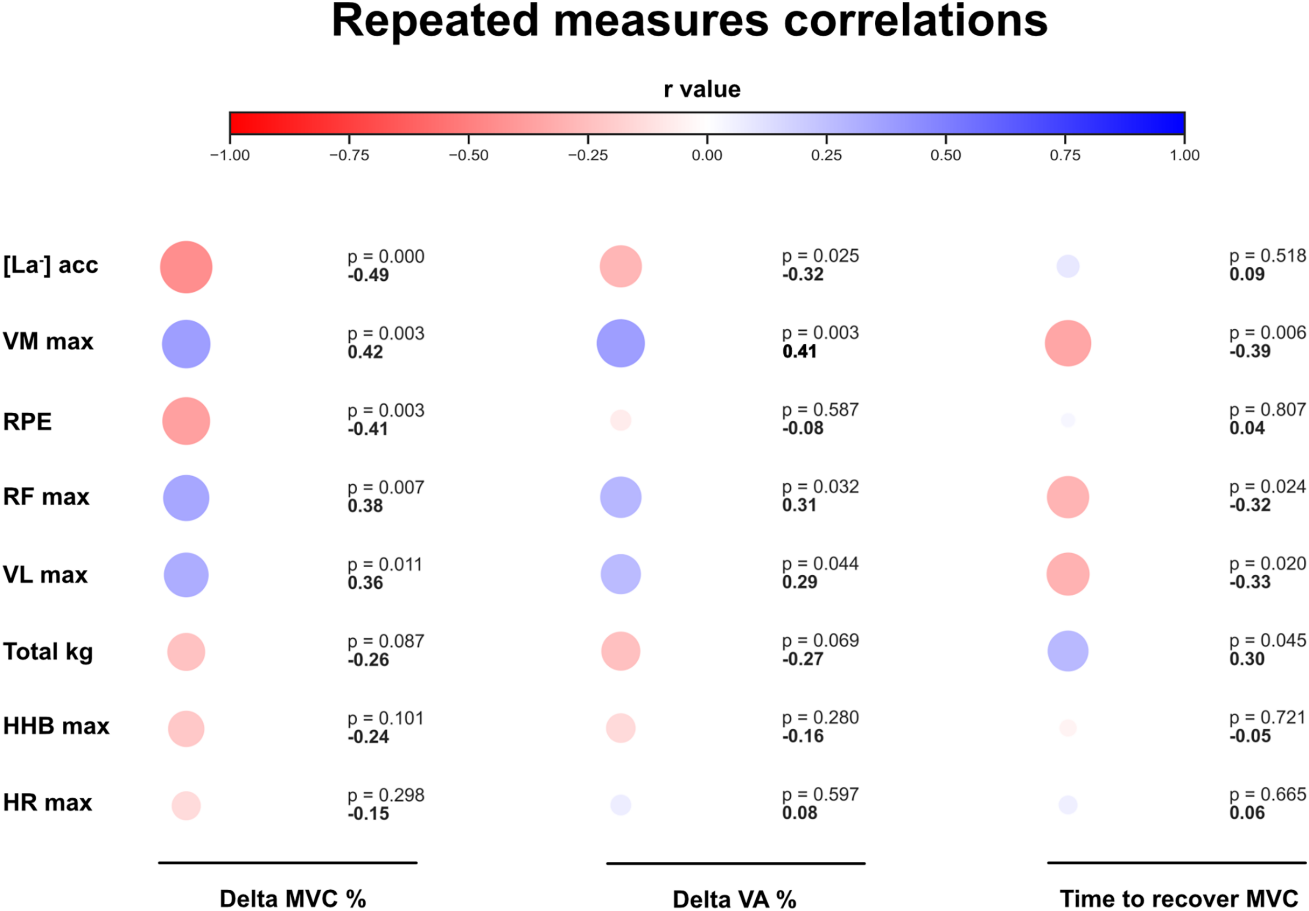
Repeated measures correlations. Repeated measures correlations (red = negative, blue = positive) between the main physiological variables measured in the study and the induced fatigue and recovery. Exact r values are reported under the *p* values.

The correlations between physiological values and fatigue are reported in Fig. 5. Finally, the ICC analysis demonstrated excellent reliability for both baseline HHB values and MVC pre-testing values across sessions. For baseline HHB, the ICC was 0.92 (95% CI [0.82–0.97], *p* < 0.001), indicating high consistency. Similarly, MVC pre-testing values showed an ICC of 0.94 (95% CI [0.87–0.98], *p* < 0.001). These findings confirm the robustness and reproducibility of the physiological measurements across testing sessions.

## Discussion

This study investigated the physiological responses and fatigue to exhaustive protocols of unilateral leg extension over the full range of % 1RM, from very low to very high external loads. Contrary to what was hypothesised, muscle failure was not reached at all % 1RM, with 10% and, partially, 30% 1RM showing behaviours that are associated with sustainable exercise. The end of the exercise was also not characterised by a common level of muscle excitation of the investigated muscles. Instead, higher loads (*i.e*., >50% 1RM) displayed higher levels of muscle excitation upon muscle failure. From a muscle metabolism perspective, similar levels of local metabolic perturbation (*i.e*., HHb) were observed at task failure across all protocols ≥30% 1RM. The same behaviour was recorded for whole-body measurements of metabolism (HR and [La^−^]) and perceived exertion. Moreover, individuals’ CL were found to lie around 31.7 ± 11.9 of the % 1RM, indicating a potential overlap of this metabolic threshold with the tension that is typically associated with occlusion of blood perfusion. Finally, in line with our last hypothesis, fatigue accumulated more at lower intensity due to the higher number of repetitions performed and higher metabolic perturbation. These findings have important implications for training designs and prescriptions and seem to suggest that muscle failure during RT occurs upon the reach of a given level of metabolic perturbation. Moreover, they could suggest a role of local metabolic thresholds (*i.e*., critical load) in the understanding of physiological responses to RT, particularly at lower % 1RM.

Previous research suggested a minimum load threshold of around 30% 1RM to elicit hypertrophic adaptations from RT (Schoenfeld, 2013; Weakley et al., 2023), due to the concomitant increase in intramuscular pressure that begins to occlude local blood flow during contraction. While such occlusion is likely attenuated in our concentric-only protocol, this threshold may still represent a transition point in local exercise intensity. By performing exercise across the repetition continuum, we were able to determine distinct acute physiological responses across loads. Specifically, muscle failure was reached by all participants during the 50%, 70%, and 90% 1RM protocols, while only six participants interrupted exercise before completing 450 repetitions (*i.e*., the predetermined cut-off for exercise termination) at 30% 1RM, and no participant reached muscle failure during the 10% trial. These results suggest that the transition to an unsustainable intensity domain (*i.e*., severe exercise intensity) may occur at a load around 30% 1RM, depending on factors such as contraction tempo, the relative contribution of the involved muscle, and the type of contraction employed.

From a metabolic perspective, all the intensities >10% 1RM presented levels of local HHB significantly different from the 10% 1RM, suggesting that exercise was terminated upon the reach of a given level of metabolic perturbation (Fig. 2). This is consistent with previous research performed with both small and large muscle masses that associated exercise muscle failure with the depletion of muscle PCr and the accumulation of fatigue-related metabolites such as P_i_ and H^+^ (Jones et al., 2008; Black et al., 2017). Importantly, while the slopes of RMS and HHB in the vastus lateralis were strongly correlated across conditions, only HHB reached a consistent value at task failure (Fig. 2). This suggests that although neuromuscular activation progressively adapted to the loading conditions, it was the metabolic perturbation that reached a threshold coinciding with muscle failure. These findings reinforce the idea that local metabolic stress may represent the limiting factor across a wide range of RT intensities. Moreover, in combination with existing literature advocating near-failure training, they support the notion that metabolic perturbation could serve as an auxiliary pathway to stimulate hypertrophic adaptations with lower-load RT. This difference between loads seemed confirmed by whole-body markers of metabolism ([La^−^] and HR), and perceived effort (Fig. 3).

A different behaviour was displayed by the muscle excitation of the VL, VM, and RF (Fig. 2). In these three muscles, the RMS values recorded upon failure seemed to increase with increasing load (Fig. 2), and did not reach a common “critical level” as might be expected from progressive fibre recruitment (Grassi, Rossiter & Zoladz, 2015). Within the limitations of the EMG technique in assessing muscle activation (Vigotsky et al., 2018) our study seems to corroborate previous research that found that muscle failure in RT does not correspond to maximal muscle activation when submaximal loads are used (Schoenfeld et al., 2014; Morton et al., 2019). Notably, we observed submaximal EMG amplitudes both in protocols where failure was reached (*e.g*., 50% and 70% 1RM, and in some 30% cases) and in conditions where failure was not reached (10% and the remaining 30%). This consistent submaximal excitation across light and moderate loads reinforces the idea that muscle failure under these conditions may result from accumulating peripheral fatigue or inhibitory feedback mechanisms rather than from complete motor unit recruitment.

Although the application of the CL concept to RT has some limitations, as many factors can affect its determination (*e.g*., exercise modalities, muscle mass involved, tempo, *etc*.), the 10% 1RM intensity, and 30% 1RM in some participants, displayed behaviours typical of sustainable exercise (Jones et al., 2019) (Figs. 2 and 3). In the framework of dynamic exercise, the CL reflects the boundary between heavy-sustainable and severe-unsustainable intensity domains. While loads ≥30% 1RM are already known to induce ischemic conditions that may mediate hypertrophic adaptations in low-load RT (Fliss et al., 2025), the present findings suggest a potential role for “local exercise domains” in shaping physiological responses during RT. Current literature places the minimal effective load for hypertrophic adaptations around 30% 1RM (Weakley et al., 2023), yet no clear physiological rationale or method exists to define this boundary at the individual level. Future research should explore whether local CL could serve as this threshold, particularly in complex, multi-joint exercises where mechanical and metabolic demands are unequally distributed between muscles.

It is also worth noting that the number of repetitions performed at 30–50% 1RM in the present study was markedly higher than studies which reported ~45 repetitions for leg extension exercise at 30% 1RM using a 1:1 concentric:eccentric tempo (Cook, Murphy & Labarbera, 2013; Akima & Saito, 2013; Jenkins et al., 2015). These differences likely reflect the impact of contraction mode and pacing on blood flow dynamics and fatigue onset. Supporting this interpretation, Fliss et al. (2025) demonstrated that up to 300 repetitions could be completed at <20% 1RM using a 2:2 concentric:eccentric tempo, reinforcing the possible role that both the absolute load and the ratio of tension time to rest between contractions play in determining local perfusion and endurance capacity in single-joint exercises (Fliss et al., 2025). To summarize, these observations suggest that the occurrence of a muscle’s CL depends not only on external load but also on contraction mode, duration, and the resulting hemodynamic environment.

To our knowledge, this is the first study to provide an extensive characterization of fatigue and recovery over the full 1RM spectrum. Despite the achievement of muscle failure in all loads ≥30% 1RM, only the 30% and 50% trials showed a significant reduction in MVC compared to baseline, and this pattern was reflected in the delta values of MVC and VA% (Fig. 3). The higher fatigue accumulated at 30% and 50% 1RM seemed due to a combination of relatively high intensity and duration of exercise. In this context, the common level of muscle perturbation (*i.e*., HHB) reached upon muscle failure, the whole-body [La^−^] accumulation, and the moderate correlation found between [La^−^] and the delta % drop in MVC (Fig. 5) support the notion that exercise was terminated when a given metabolic perturbation was reached. Overall, these results seem in line with a previous investigation performed at the biceps level, which found increased fatigue development with an increasing number of repetitions performed (up to 20RM (Behm et al., 2002)), and provide new insight into the time needed to recover from RT performed to the point of muscle failure with different loads. Within the sensitivity of our %VA measurement technique (*i.e*., single twitch interpolation), central fatigue did not appear to be a major differentiating factor between conditions, and %VA remained relatively stable across loads. Notwithstanding this, the interpretation of these results should take into account that participants performed only one set of exhaustive unilateral leg extension, and that findings (*e.g*., the drop in %VA) might differ in multi-set protocols or exercises involving larger muscle masses as these can lead to cumulative fatigue and influence both the time to failure (Nagle, Seals & Hanson, 1988; Law & Avin, 2010; Morán-Navarro et al., 2017) and the physiological responses to exercise (Ratamess et al., 2014).

**Figure 6:**
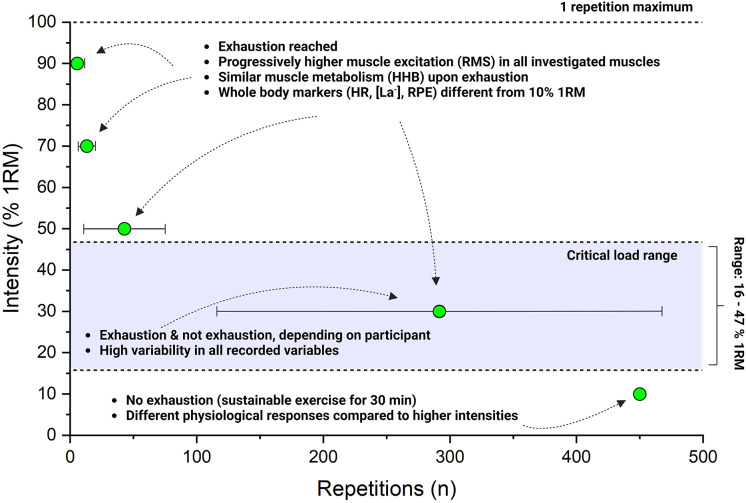
Schematic representation of the physiological responses reported in the study at different % of the one repetition maximum.

This study provides important insight into the acute responses to RT performed with different % 1RM (as resumed in Fig. 6). The novelty and the practical implications of this study, if confirmed by further research, would be: (i) the attainment of a common level of metabolic perturbation upon muscle failure in conditions ≥30% 1RM, noting that this was not observed at 10% 1RM (ii) confirming the importance of % 1RM in determining the maximal levels of muscle excitation reached upon muscle failure, which does not reach maximal values at all loads due to progressive recruitment of muscle fibres (iii) the possible detection of the minimum threshold for RT load in concomitance of the exercise’s CL (iv) the relationship between fatigue during RT and metabolic perturbation.

Moreover, this research may also inform future investigations or clinical RT programs. In particular, the identification of a potential metabolic threshold for task failure at moderate loads could support the design of low-load protocols for undertrained or rehabilitative populations, where heavier loads may be contraindicated or less tolerated (Colosio et al., 2025). Within the protocol utilised and the healthy population investigated, the present results suggest that using loads above the estimated CL, roughly corresponding to ≥30% 1RM and performed close to failure, as typically recommended, may be necessary to reach a common level of metabolic perturbation and thus ensure an adequate training stimulus.

## Limitations

It should be acknowledged that the physiological responses documented at the muscle level are only representative of the exercise model employed, which involved concentric-only unilateral leg extensions at a controlled tempo and performed on a machine with a predefined strength curve (Fig. S1) and without pre-imposing contraction duration between participants and loads. As such, care should be taken when generalising these findings to other RT modes that involve different mechanical characteristics or include eccentric phases or different timing.

Moreover, the physiological responses documented at the muscle level are only representative of the investigated muscle areas and might differ in other muscle regions. The main power analysis was based on MVC, and the sample size is consistent with similar repeated-measures studies in physiology. However, statistical power may be lower for variables with higher signal-to-noise ratios, such as EMG. The utilised technique for electrical stimulation (*i.e*., singlets and not bursts of doublets, triplets, *etc*.) might have increased the noise of the measure and potentially masked some drop in %VA (Shield & Zhou, 2004; Osborne et al., 2023). Although this choice was taken to limit participants’ discomfort during repetitive MVCs (Shield & Zhou, 2004), this methodological aspect should be considered in the context of the lack of difference in %VA between % 1RM. Nevertheless, the excellent reliability of baseline MVCs (ICC > 0.95) strengthens confidence in the observed force reductions as genuine markers of fatigue, even if the precise central contribution remains challenging to isolate.

Although this study was not specifically aimed at investigating differences between biological sexes, it is worth noting that five out of the six participants who exhibited a CL above 30% 1RM were women (women: 36 ± 14% 1RM *vs*. men: 28 ± 9% 1RM). While we believed that the main effect in the present study was increased variability in the responses during the 30% 1RM protocol, this trend suggests that underlying physiological factors (*e.g*., fibre type composition (Nuzzo, 2024)) may influence responses to low-load RT differently in men and women. Future studies should be explicitly powered and structured to test for sex-based interactions in this context. Finally, we acknowledge that contraction time and inter-repetition rest were not strictly fixed, as these varied slightly across loads and progressively with fatigue. While brief rest periods did occur between repetitions, they were considered a minor limitation compared to the risk of inducing continuous tension, which could have led to local ischemia and artificially shortened exercise duration, especially at lower loads. Our protocol introduced a fixed repetition frequency designed to minimise variability in order to isolate the effect of different loading conditions while avoiding secondary confounding factors inherent to self-paced RT.

## Conclusions

This study gives insight into the acute responses to concentric-only, single-joint RT across different % 1RM. The findings highlight a possible common level of metabolic perturbation with loads of ≥30% 1RM, contrasting with lower loads. % 1RM seems an important determinant of maximal muscle excitation levels upon muscle failure, and we potentially identified the minimum RT load threshold in coincidence with the exercise’s CL. Furthermore, we observed a relationship between fatigue and metabolic perturbation that might have important implications when designing low-load RT. These findings have practical implications for exercise prescription, aiding practitioners in balancing training stimuli and managing fatigue across different % 1RM.

## Supplemental Information

10.7717/peerj.20909/supp-1 Supplemental Information 1 Force-displacement profiles of a representative participant performing unilateral concentric leg extensions at 30%, 50%, and 70% of 1RM. Force was measured with a linear encoder positioned at the footpad. The curves illustrate the characteristic resistance pattern (mean of 3 repetitions) of the machine across the range of motion.

10.7717/peerj.20909/supp-2 Supplemental Information 2 Representative HHB and EMG data from a representative participant across five intensities (10–90% 1RM). Note the relatively stable profile at low loads and the progressive increase and convergence toward higher HHB values at higher loads terminating in failure.

10.7717/peerj.20909/supp-3 Supplemental Information 3 Repeated measures correlations between the main physiological variables measured in the study and the induced fatigue and recovery. Exact r values are reported under the p values. Note: delta values are expressed as negative (*e.g*., higher lactate accumulation is associated with a greater reduction in MVC).

10.7717/peerj.20909/supp-4 Supplemental Information 4 All the data from the articles and additional supplementary data on the NIRS and EMG signals.

## Additional Information and Declarations

### Data Availability

The following information was supplied regarding data availability:

The raw data is available in the Supplemental Files.

## References

[ref-1] Akima H, Saito A (2013). Activation of quadriceps femoris including vastus intermedius during fatiguing dynamic knee extensions. European Journal of Applied Physiology.

[ref-2] Bakdash JZ, Marusich LR (2017). Repeated measures correlation. Frontiers in Psychology.

[ref-3] Behm DG, Reardon G, Fitzgerald J, Drinkwater E (2002). The effect of 5, 10, and 20 repetition maximums on the recovery of voluntary and evoked contractile properties. Journal of Strength and Conditioning Research.

[ref-4] Black MI, Jones AM, Blackwell JR, Bailey SJ, Wylie LJ, McDonagh STJ, Thompson C, Kelly J, Sumners P, Mileva KN, Bowtell JL, Vanhatalo A (2017). Muscle metabolic and neuromuscular determinants of fatigue during cycling in different exercise intensity domains. Journal of Applied Physiology.

[ref-5] Brzycki M (1993). Predicting a one-rep max from reps-to-fatigue. Journal of Physical Education, Recreation and Dance.

[ref-6] Burd NA, West DWD, Staples AW, Atherton PJ, Baker JM, Moore DR, Holwerda AM, Parise G, Rennie MJ, Baker SK, Phillips SM (2010). Low-load high volume resistance exercise stimulates muscle protein synthesis more than high-load low volume resistance exercise in young men. PLoS ONE.

[ref-7] Caen K, Bourgois JG, Bourgois G, Van Der Stede T, Vermeire K, Boone J (2019). The reconstitution of W’ depends on both work and recovery characteristics. Medicine and Science in Sports and Exercise.

[ref-8] Cannon J, Marino FE (2010). Early-phase neuromuscular adaptations to high- and low-volume resistance training in untrained young and older women. Journal of Sports Sciences.

[ref-9] Colosio AL, Caen K, Bourgois JG, Boone J, Pogliaghi S (2021). Metabolic instability vs fibre recruitment contribution to the V˙O_2_ slow component in different exercise intensity domains. Pflugers Archiv European Journal of Physiology.

[ref-10] Colosio AL, Pogliaghi S (2020). Investigating the physiological mechanisms of the oxygen consumption “slow component”. PhD thesis. University Of Verona, Verona.

[ref-11] Colosio AL, Teso M, Bottari A, Ferrari L, Bochicchio G, Boone J, Pogliaghi S (2025). Cardiocirculatory and metabolic responses to low- and high-load squat exercise in young and middle-aged individuals. Journal of Functional Morphology and Kinesiology.

[ref-12] Cook SB, Murphy BG, Labarbera KE (2013). Neuromuscular function after a bout of low-load blood flow-restricted exercise. Medicine and Science in Sports and Exercise.

[ref-13] Farina D (2006). Interpretation of the surface electromyogram in dynamic contractions. Exercise and Sport Sciences Reviews.

[ref-14] Fliss MD, Abercrombie MJ, Denson KG, Wiens L, Losciale JM, Schweitzer AM, Coccimiglio IF, Tripp TR, Burr JF, MacInnis MJ, Mitchell CJ (2025). A critical occluding tension phase transition occurs between 30% and 40% 1RM in dynamic knee extension exercise. Scandinavian Journal of Medicine and Science in Sports.

[ref-15] Grassi B, Quaresima V (2016). Near-infrared spectroscopy and skeletal muscle oxidative function in vivo in health and disease: a review from an exercise physiology perspective. Journal of Biomedical Optics.

[ref-16] Grassi B, Rossiter HB, Zoladz JA (2015). Skeletal muscle fatigue and decreased efficiency: two sides of the same coin?. Exercise and Sport Sciences Reviews.

[ref-17] Henneman E, Somjen G, Carpenter DO (1965). Excitability and inhibitibility of motoneurons of different sizes. Journal of Neurophysiology.

[ref-18] Hermens HJ, Freriks B, Disselhorst-Klug C, Rau G (2000). Development of recommendations for SEMG sensors and sensor placement procedures. Journal of Electromyography and Kinesiology.

[ref-19] Inglis EC, Iannetta D, Rasica L, Mackie MZ, Keir DA, Macinnis MJ, Murias JM (2024). Heavy-, severe-, and extreme-, but not moderate-intensity exercise increase V˙O_2_max and thresholds after 6 wk of training. Medicine and Science in Sports and Exercise.

[ref-20] Jackson A, Blair S, Mahar MT, Wier LT, Ross RT, Stuteville JE (1990). Prediction of functional aerobic capacity without exercise testing. Medicine and Science in Sports and Exercise.

[ref-21] Jenkins NDM, Housh TJ, Bergstrom HC, Cochrane KC, Hill EC, Smith CM, Johnson GO, Schmidt RJ, Cramer JT (2015). Muscle activation during three sets to failure at 80 vs. 30% 1RM resistance exercise. European Journal of Applied Physiology.

[ref-22] Jones AM, Burnley M, Black MI, Poole DC, Vanhatalo A (2019). The maximal metabolic steady state: redefining the ‘gold standard’. Physiological Reports.

[ref-23] Jones AM, Wilkerson DP, DiMenna F, Fulford J, Poole DC (2008). Muscle metabolic responses to exercise above and below the critical power assessed using 31P-MRS. American Journal of Physiology-Regulatory, Integrative and Comparative Physiology.

[ref-24] Kadlec D, Jordan MJ, Snyder L, Alderson J, Nimphius S (2021). Test re-test reliability of single and multijoint strength properties in female australian footballers. Sports Medicine—Open.

[ref-25] Kraemer WJ, Ratamess NA, Flanagan SD, Shurley JP, Todd JS, Todd TC (2017). Understanding the science of resistance training: an evolutionary perspective. Sports Medicine.

[ref-26] Lasevicius T, Schoenfeld BJ, Silva-Batista C, de Barros TS, Aihara AY, Brendon H, Longo AR, Tricoli V, de Peres BA, Teixeira EL (2022). Muscle failure promotes greater muscle hypertrophy in low-load but not in high-load resistance training. Journal of Strength and Conditioning Research.

[ref-27] Lasevicius T, Ugrinowitsch C, Schoenfeld BJ, Roschel H, Tavares LD, De Souza EO, Laurentino G, Tricoli V (2018). Effects of different intensities of resistance training with equated volume load on muscle strength and hypertrophy. European Journal of Sport Science.

[ref-28] Law LAF, Avin KG (2010). Endurance time is joint-specific: a modelling and meta-analysis investigation. Ergonomics.

[ref-29] Lawson D, Vann C, Schoenfeld BJ, Haun C (2022). Beyond mechanical tension: a review of resistance exercise-induced lactate responses & muscle hypertrophy. Journal of Functional Morphology and Kinesiology.

[ref-30] Loenneke JP, Wilson GJ, Wilson JM (2010). A mechanistic approach to blood flow occlusion. International Journal of Sports Medicine.

[ref-31] Maden-Wilkinson TM, Balshaw TG, Massey GJ, Folland JP (2020). What makes long-term resistance-trained individuals so strong? A comparison of skeletal muscle morphology, architecture, and joint mechanics. Journal of Applied Physiology.

[ref-32] Monod H, Scherrer J (1965). The work capacity of a synergic muscular group. Ergonomics.

[ref-33] Morton RW, Sonne MW, Farias Zuniga A, Mohammad IYZ, Jones A, McGlory C, Keir PJ, Potvin JR, Phillips SM (2019). Muscle fibre activation is unaffected by load and repetition duration when resistance exercise is performed to task failure. The Journal of Physiology.

[ref-34] Morán-Navarro R, Pérez CE, Mora-Rodríguez R, de la Cruz-Sánchez E, González-Badillo JJ, Sánchez-Medina L, Pallarés JG (2017). Time course of recovery following resistance training leading or not to failure. European Journal of Applied Physiology.

[ref-35] Nagle FJ, Seals DR, Hanson P (1988). Time to fatigue during isometric exercise using different muscle masses. International Journal of Sports Medicine.

[ref-36] Nuzzo JL (2024). Sex differences in skeletal muscle fiber types: a meta-analysis. Clinical Anatomy.

[ref-37] Ogborn D, Schoenfeld BJ (2014). The role of fiber types in muscle hypertrophy: implications for loading strategies. Strength and Conditioning Journal.

[ref-38] Osborne JO, Tallent J, Girard O, Marshall PW, Kidgell D, Buhmann R (2023). Neuromuscular electrical stimulation during maximal voluntary contraction: a Delphi survey with expert consensus. European Journal of Applied Physiology.

[ref-39] Pogliaghi S, Teso M, Ferrari L, Boone J, Murias JM, Colosio AL (2023). Easy prediction of the maximal lactate steady-state in young and older men and women. Journal of Sports Science and Medicine.

[ref-40] Ratamess N, Alvar B, Evetoch T, Housh T, Kibler W, Kraemer W, Triplett N (2009). Progression models in resistance training for healthy adults. Medicine and Science in Sport and Exercise.

[ref-41] Ratamess NA, Rosenberg JG, Klei S, Dougherty BM, Kang J, Smith CR, Ross RE, Faigenbaum AD (2014). Acute oxygen uptake and resistance exercise performance using different rest interval lengths: the influence of maximal aerobic capacity and exercise sequence. Journal of Strength and Conditioning Research.

[ref-42] Robertson RJ, Goss FL, Rutkowski J, Lenz B, Dixon C, Timmer J, Frazee K, Dube J, Andreacci J (2003). Concurrent validation of the OMNI perceived exertion scale for resistance exercise. Medicine and Science in Sports and Exercise.

[ref-43] Schoenfeld BJ (2013). Is there a minimum intensity threshold for resistance training-induced hypertrophic adaptations?. Sports Medicine.

[ref-44] Schoenfeld BJ, Contreras B, Willardson JM, Fontana F, Tiryaki-Sonmez G (2014). Muscle activation during low- versus high-load resistance training in well-trained men. European Journal of Applied Physiology.

[ref-45] Schoenfeld BJ, Grgic J, Ogborn D, Krieger JW (2017). Strength and hypertrophy adaptations between low- vs. high-load resistance training: a systematic review and meta-analysis. Journal of Strength and Conditioning Research.

[ref-46] Schoenfeld BJ, Grgic J, Van Every DW, Plotkin DL (2021). Loading recommendations for muscle strength, hypertrophy, and local endurance: a re-examination of the repetition continuum. Sports.

[ref-47] Shield A, Zhou S (2004). Assessing voluntary muscle activation with the twitch interpolation technique. Sports Medicine.

[ref-48] Strojnik V, Komi PV (1998). Neuromuscular fatigue after maximal stretch-shortening cycle exercise. Journal of Applied Physiology.

[ref-49] Stuer L, Teso M, Colosio AL, Loi M, Mucci P, Pogliaghi S, Boone J, Caen K (2024). The impact of skinfold thickness and exercise intensity on the reliability of NIRS in the vastus lateralis. European Journal of Applied Physiology.

[ref-50] Twomey R, Aboodarda SJ, Kruger R, Culos-Reed SN, Temesi J, Millet GY (2017). Neuromuscular fatigue during exercise: methodological considerations, etiology and potential role in chronic fatigue. Neurophysiologie Clinique.

[ref-51] Vigotsky AD, Halperin I, Lehman GJ, Trajano GS, Vieira TM (2018). Interpreting signal amplitudes in surface electromyography studies in sport and rehabilitation sciences. Frontiers in Physiology.

[ref-52] Wackerhage H, Schoenfeld BJ, Hamilton DL, Lehti M, Hulmi JJ (2019). Stimuli and sensors that initiate skeletal muscle hypertrophy following resistance exercise. Journal of Applied Physiology.

[ref-53] Weakley J, Schoenfeld BJ, Ljungberg J, Halson SL, Phillips SM (2023). Physiological responses and adaptations to lower load resistance training: implications for health and performance. Sports Medicine—Open.

[ref-54] Williamson A, Hoggart B (2005). Pain: a review of three commonly used pain rating scales. Journal of Clinical Nursing.

