# Supplementary figures and images for: Minimum load threshold in resistance training: insights into muscle metabolism, excitation, and fatigue across the repetition continuum
Source: PeerJ. 2026 Mar 12;14:e20909. doi: 10.7717/peerj.20909 (PMC12989152; doi:10.7717/peerj.20909)

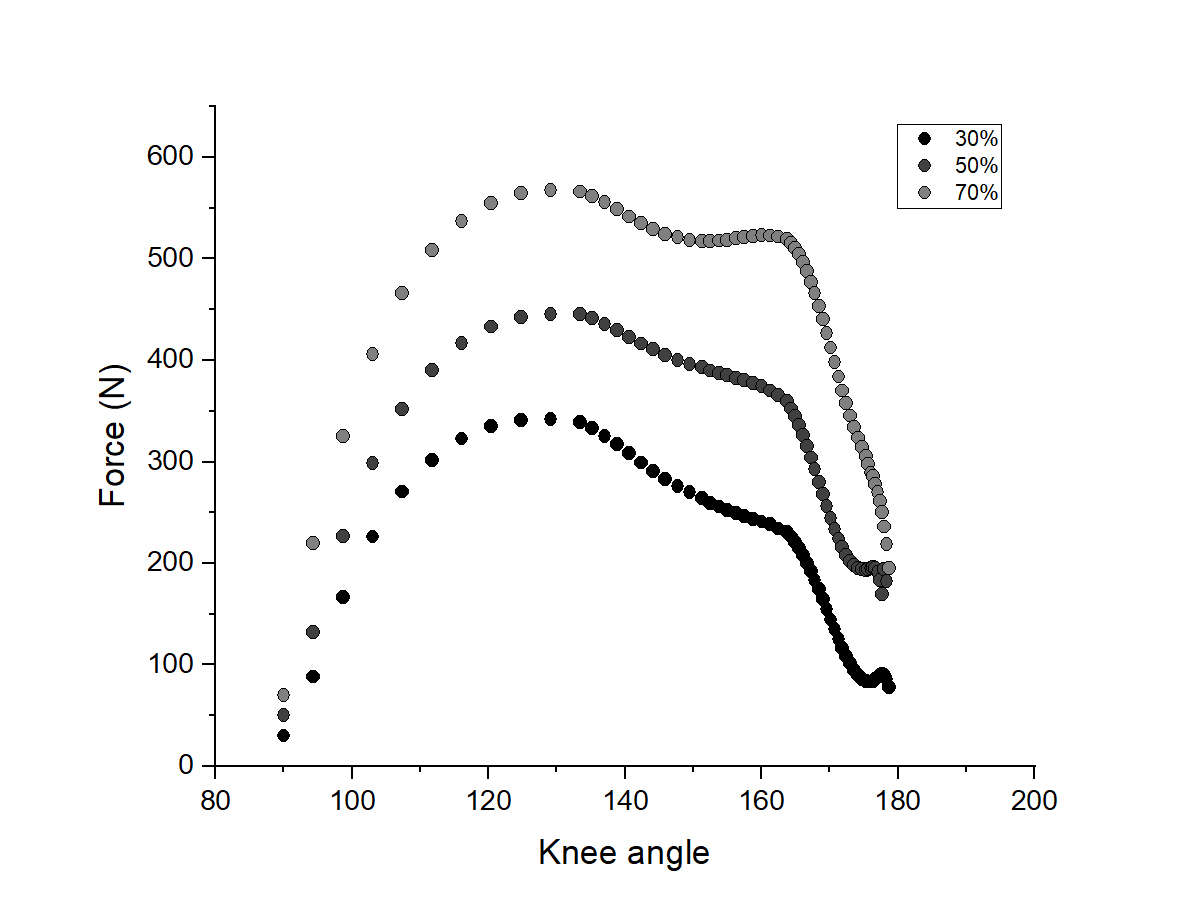

Supplement: Supplemental Information 1 — Force was measured with a linear encoder positioned at the footpad. The curves illustrate the characteristic resistance pattern (mean of 3 repetitions) of the machine across the range of motion. [file peerj-14-20909-s001.jpg]

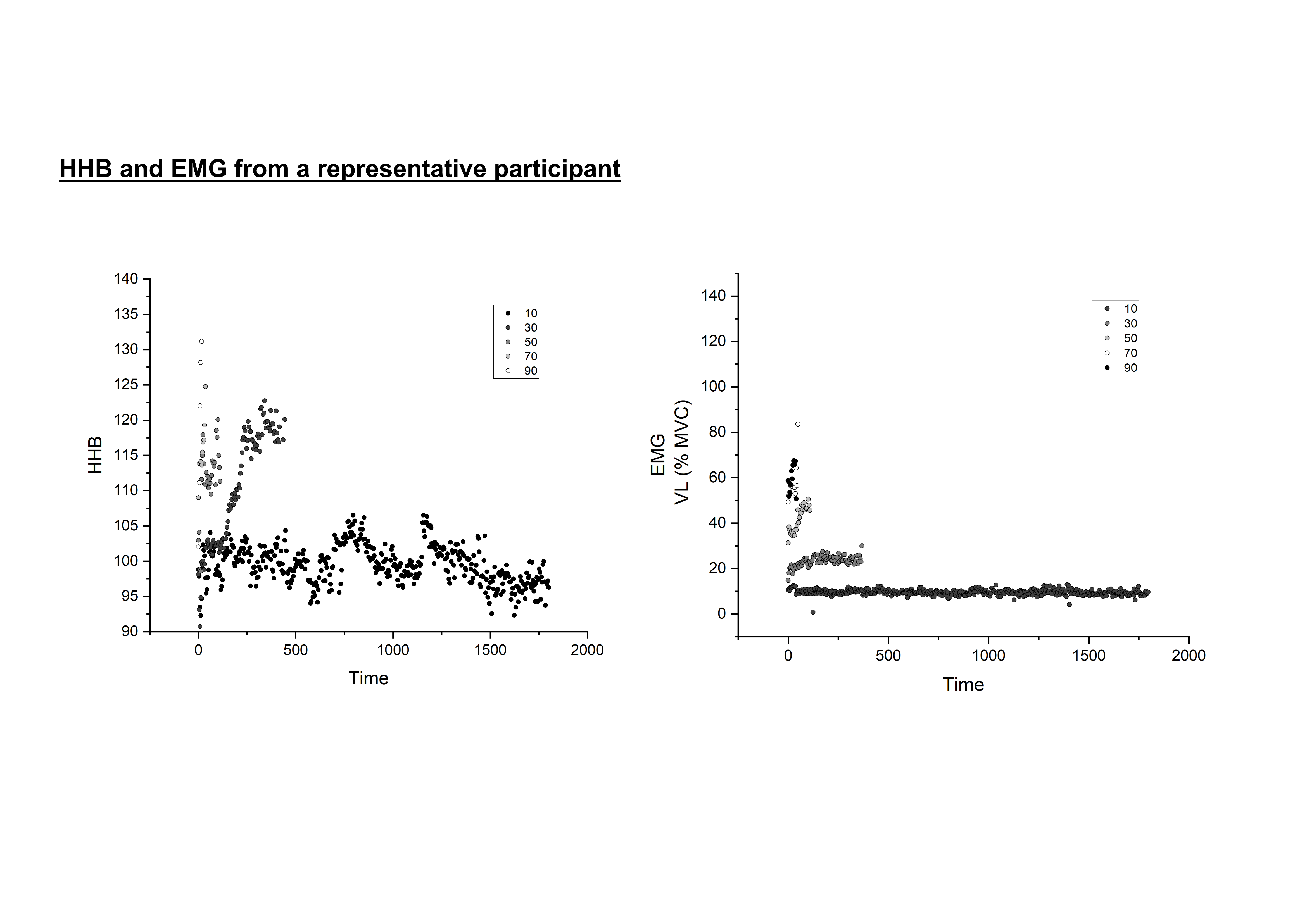

Supplement: Supplemental Information 2 — Note the relatively stable profile at low loads and the progressive increase and convergence toward higher HHB values at higher loads terminating in failure. [file peerj-14-20909-s002.png]
