# Supplementary material for: Minimum load threshold in resistance training: insights into muscle metabolism, excitation, and fatigue across the repetition continuum
Source: PeerJ. 2026 Mar 12;14:e20909. doi: 10.7717/peerj.20909 (PMC12989152; doi:10.7717/peerj.20909)
Supplement: Supplemental Information 3 — Exact r values are reported under the p values. Note: delta values are expressed as negative (e.g., higher lactate accumulation is associated with a greater reduction in MVC). [file peerj-14-20909-s003.docx]

**Supplementary Table – Repeated Measures Correlations**

| **Variable** | **Delta MVC % (r)** | **Delta MVC % (p)** | **Delta VA % (r)** | **Delta VA % (p)** | **Time to recover MVC (r)** | **Time to recover MVC (p)** |
| --- | --- | --- | --- | --- | --- | --- |
| **[La⁻] acc** | -0.49 | 0.000 | -0.32 | 0.025 | 0.09 | 0.518 |
| **VM max** | 0.42 | 0.003 | 0.41 | 0.003 | -0.39 | 0.006 |
| **RPE** | -0.41 | 0.003 | -0.08 | 0.587 | 0.04 | 0.807 |
| **RF max** | 0.38 | 0.007 | 0.31 | 0.032 | -0.32 | 0.024 |
| **VL max** | 0.36 | 0.011 | 0.29 | 0.044 | -0.33 | 0.020 |
| **Total kg** | -0.26 | 0.087 | -0.27 | 0.069 | 0.30 | 0.045 |
| **HHB max** | -0.24 | 0.101 | -0.16 | 0.280 | -0.05 | 0.721 |
| **HR max** | -0.15 | 0.298 | 0.08 | 0.597 | 0.06 | 0.665 |
